# RGB Imaging and Irrigation Management Reveal Water Stress Thresholds in Three Urban Shrubs in Northern China

**DOI:** 10.3390/plants14152253

**Published:** 2025-07-22

**Authors:** Yuan Niu, Xiaotian Xu, Wenxu Huang, Jiaying Li, Shaoning Li, Na Zhao, Bin Li, Chengyang Xu, Shaowei Lu

**Affiliations:** 1 Urban Forestry Research Center, Beijing Forestry University, Beijing 100083, China; ny652301@163.com; 2 Institute of Forestry and Pomology, Beijing Academy of Agriculture and Forestry Sciences, Beijing 100093, China; xuxiaotian@baafs.net.cn (X.X.); huangwx202206@163.com (W.H.); lijiaying6688@126.com (J.L.); lishaoning@baafs.net.cn (S.L.); zhaona@baafs.net.cn (N.Z.); sxdxlibin@126.com (B.L.); 3 Beijing Yanshan Forest Ecosystem Research Station, National Forest and Grassland Administration, Beijing 100093, China; 4 College of Forestry, Shenyang Agricultural University, Shenyang 110161, China

**Keywords:** image processing, color mode, drought tolerance, color index

## Abstract

The context of global climate change, water stress has a significant impact on the ecological function and landscape value of urban greening shrubs. In this study, three typical greening shrubs (*Euonymus japonicus*, *Ligustrum* × *vicaryi*, and *Berberis thunbergii* var. *atropurpurea*) in North China were subjected to a two-year field-controlled experiment (2022–2023) with four water treatments: full irrigation, deficit irrigation, natural rainfall, and extreme drought. The key findings are as follows: (1) Extreme drought reduced the color indices substantially—the G_CC_ of *E. japonicus* decreased by 40% (2023); the R_CC_ of *B. thunbergii* var. *atropurpurea* declined by 35% (2022); and the color indices of *L.* × *vicaryi* remained stable (variation < 15%). (2) Early-season soil water content (SWC) strongly correlated with the color index of *E. japonicus* (r^2^ = 0.42, *p* < 0.05) but weakly with *B. thunbergii* (r^2^ = 0.28), suggesting species-specific drought-tolerance mechanisms like reduced leaf area. (3) Deficit irrigation (SWC ≈ 40%) maintained color indices between fully irrigated and drought-stressed levels. Notably, *B. thunbergii* retained high redness (R_CC_ > 0.8) at an SWC ≈ 40%; *E. japonicus* required an SWC > 60% to preserve greenness (G_CC_). The research results provide a scientific basis for urban greening plant screening and water-saving irrigation strategies, and expand the application scenarios of color coordinates in plant physiological and ecological research.

## 1. Introduction

With global climate change and accelerated urbanization, water scarcity has become a key bottleneck constraining sustainable urban development [1]. As an important component of urban ecosystems, greening shrubs provide a variety of ecological service functions for human beings through processes such as carbon fixation and heat island effect mitigation [2], and their physiological response mechanisms to environmental changes and landscape performance are hot spots in urban ecology research. Plant color influences residents’ emotional states through neuroendocrine regulatory mechanisms and is a critical aspect of landscape aesthetics [3]. Water stress, i.e., inadequate water supply—as a common environmental stress—decreases the rate of photosynthesis in plants, affects root growth and nutrient uptake, and consequently affects plant growth, development, physiology, and metabolism. Dynamic changes in plant leaf color are closely related to physiological and metabolic processes, and water stress can induce changes in pigment synthesis pathways, which in turn affect the ecological function and landscape value of greening shrubs [4]. In some ornamental shrubs (e.g., heather *Photinia* × *fraseri*), water stress can produce more vibrant autumn coloration without affecting biomass accumulation [5]. This finding provides a theoretical basis for developing a precision irrigation regime based on plant-water-response thresholds [6].

Changes in plant leaf color are not only controlled by genes but also by environmental factors such as climate, light, temperature, humidity, and soil conditions [7,8]. These factors influence plant color expression by altering the pigment composition of leaves, such as chlorophyll, carotenoids, and anthocyanins [9]. In particular, water stress leads to a decrease in the water content of plant leaves, which in turn affects the photosynthesis and pigmentation content of the leaves, ultimately leading to changes in plant color. For example, a study by Chalker-Scott (1999) found that reduced water leads to accelerated chlorophyll degradation in the leaves of sorghum and purple-leaved dwarf cherry, giving the leaves a yellow or brown color [10], and a study by Gill et al. (2015) pointed out that under drought conditions, the anthocyanin content of certain plants increases, giving the leaves a red or purple color, which is a helpful adaptive mechanism of plants to reduce light damage [11]. However, not all studies have shown that water stress significantly changes the color of plant leaves. For example, a study by Dabravolski et al. (2023) found that certain ornamental plants did not undergo significant changes in leaf color under mild water stress, showing strong drought tolerance [12]. This suggests that the response and adaptation of leaf color to arid environments need to be considered comprehensively when selecting urban greening plants, thus providing guidance for the development of rational irrigation regimes.

At present, the main indicators used in the research on leaf color include leaf color parameters—such as hue, saturation, and lightness—as well as RGB values (red, green, and blue) and chlorophyll content [13,14,15,16,17,18]. These indicators are widely used to quantify leaf color changes and their relationships to environmental factors and plant health. For example, a study on plants in the Mediterranean region found that leaf brightness and saturation reflect plant adaptation to arid environments, and that plants with brighter leaves usually have greater reflective capacity to reduce water evaporation [19]. A study by Gitelson et al. stated that the quantitative analysis of plant leaf color using RGB values revealed that RGB values were effective in distinguishing the leaf color of different species of plants and were significantly correlated with chlorophyll content [18]. Chlorophyll content is an important factor affecting leaf color, and many studies have inverted chlorophyll content by spectral analysis or RGB values. A study by Ustin et al. used hyperspectral imaging to model the quantitative relationship between chlorophyll content and leaf color parameters [20], and found that leaf color parameters (e.g., hue and saturation) were closely related to photosynthetic efficiency. In a study by Poorter et al., it was found that plants with darker-colored leaves usually have higher photosynthetic efficiency, which is related to the chlorophyll content and leaf structure [21]. However, most existing studies focus on agricultural crops or economic forest trees (e.g., rice, wheat, and apple trees), and while some studies have shown that the use of green and red color coordinates can reflect the response of plant phenology to environmental changes [22,23], few have reported on their application in evaluating the ornamental properties of greening plants. In addition, existing leaf color studies mostly focus on tall trees (e.g., oak and pine); the study of greening shrubs in an urban environment is very limited, and there is a lack of quantitative research on the effect of water stress on the color index of greening shrubs, which limits our comprehensive understanding of color change patterns in greening shrubs under water stress. Furthermore, interannual climatic fluctuations may modulate the impacts of drought stress on plant color dynamics, necessitating integrated assessments of phenological and environmental drivers.

This study evaluated how water stress affects leaf color—and thus ornamental value—in three common urban greening shrubs (*Euonymus japonicus*, *Ligustrum* × *vicaryi*, and *Berberis thunbergii* var. *atropurpurea*) of North China. Through controlled irrigation treatments (full irrigation/deficit irrigation/natural rainfall/extreme drought), we quantified drought-induced changes in color indices (G_CC_, Y_CC_, and R_CC_) and analyzed their relationships with the soil water content (SWC) and plant traits. Our findings establish species-specific water stress thresholds and drought-adaptation mechanisms, providing a scientific basis for water-efficient urban landscaping and shrub selection.

## 2. Results

### 2.1. Color Indices of Plants Under Full Irrigation Condition

**Figure 1:**
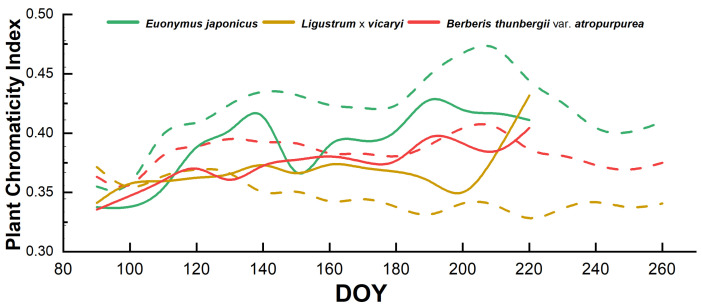
Seasonal changes in plant colorimetric index under adequate irrigation condition—dashed line indicates 2022, solid line indicates 2023; DOY: day of year.

Figure 1 illustrates the seasonal changes in leaf coloration of the three shrubs under the fully irrigated (i.e., no drought stress) condition. In both years, the green color index of *E. japonicus* was low at the beginning of the year, then gradually increased—peaking at mid-year (10 June) (0.414)—and then declined again, with a higher peak in 2023 than in 2022, with greener foliage. The yellow color index of *L*. × *vicaryi* followed a similar trend to that of large-leaved boxwood over the two years but peaked slightly lower in 2023 than in 2022. The red color index of *B*. *thunbergii* var. *atropurpurea* varied relatively little between the two years (0.347 to 0.419) but peaked significantly higher in 2023 than in 2022. These changes reflect the physiological cycles of plants, such as the growth of new leaves in spring and the senescence of leaves in fall. From the pattern of changes in their color indices, it can be seen that the primary color chromaticity indices of different plant species respond differently to the natural changes in the environment.

### 2.2. Changes in Plant Coloration and Response Under Different Treatments

**Figure 2:**
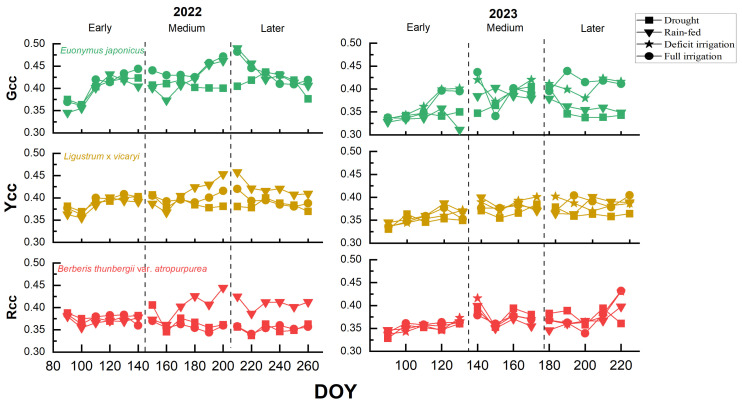
Changes in plant color index of three different tree species (*E. japonicus*, *L*. × *vicaryi*, *B*. *thunbergii* var. *atropurpurea*) under different moisture treatment conditions in 2022 and 2023.

Throughout the two experimental years with the three treatment periods, the plants had the highest primary color chromaticity indices (G_CC_, Y_CC_, and R_CC_) under the fully irrigated condition (values of 0.439–0.471, 0.404–0.419, and 0.369–0.431, respectively), suggesting that adequate water is essential for plant growth and photosynthesis. The Y_CC_ and R_CC_ of the plants decreased under extreme drought conditions (*p*-value of 0.002 for *B*. *thunbergii* var. *atropurpurea* in 2022; *p*-values of 0.05 and 0.02 for *L*. × *vicaryi* and *B*. *thunbergii* var. *atropurpurea* in 2023, respectively). The most significant decreases in the R_CC_ for 2022, *B*. *thunbergii* var. *atropurpurea*, and the G_CC_ for 2023, *E. japonicus* (0.347, *p*-value 0.05), were observed under the late-growing-season treatments. The Y_CC_ of *L*. × *vicaryi* did not change much under the three moisture conditions, suggesting that this yellow coloration is less sensitive to moisture changes. The G_CC_, Y_CC_, and R_CC_ of the plants under deficit irrigation conditions in 2023 were intermediate between the fully irrigated and drought conditions, suggesting that moderate water management can mitigate the effects of drought (Figure 2).

**Figure 3:**
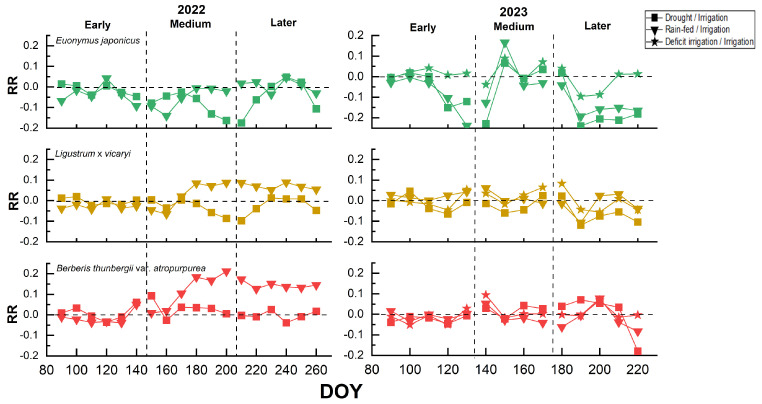
Response ratios (RR) over time (DOY, day of year) for three different shrub species (*E. japonicus*, *L.* × *vicaryi*, *B. thunbergii* var. *atropurpurea*) under different water treatment conditions in 2022 and 2023.

The colorimetric response ratios demonstrated trends in shrub leaf colorimetric indices under the reduced irrigation conditions compared to the fully irrigated condition (Figure 3). In 2022, under the drought treatment, the response ratios were mostly negative throughout the growing season, indicating that drought negatively affected the growth of *E. japonicus*, *L.* × *vicaryi*, and *B. thunbergii* var. *atropurpurea*, with *E. japonicus* showing the largest decline (lowest near −0.17) and *B. thunbergii* var. *atropurpurea* showing the smallest decline (0.01); however, under rain-fed conditions, the Y_CC_ of *L.* × *vicaryi* and the R_CC_ of *B. thunbergii* var. *atropurpurea* in the mid-to-late growing season were significantly higher than those of the fully irrigated group (RR > 0.06). In 2023, the response ratios of the Y_CC_ for *L.* × *vicaryi* and R_CC_ for *B. thunbergii* var. *atropurpurea* fluctuated less overall, indicating that the changes in irrigation had little effect (except for the drought group in the late growing season, which was close to −0.20), whereas the response of the G_CC_ of *E. japonicus* was more sensitive than in 2022—where there was a positive effect of a transient increase in response ratios in the middle of the growing season (with changes of −0.01 to 0.09); the response ratio of the deficit-irrigation group had a response ratio close to 0.00, indicating little difference from the fully irrigated group. Overall, the G_CC_ of *E. japonicus* was the most sensitive to moisture changes, while reduced irrigation had little effect or even promoted the Y_CC_ of *L.* × *vicaryi* and the R_CC_ of *B. thunbergii* var. *atropurpurea*. In addition, the data from 2022 and 2023 indicated that the year may influence the effect of the moisture treatments, which may be related to climatic conditions, soil moisture, and other factors.

### 2.3. Factors Influencing the Degree of Coloration in Plant Leaves

**Figure 4:**
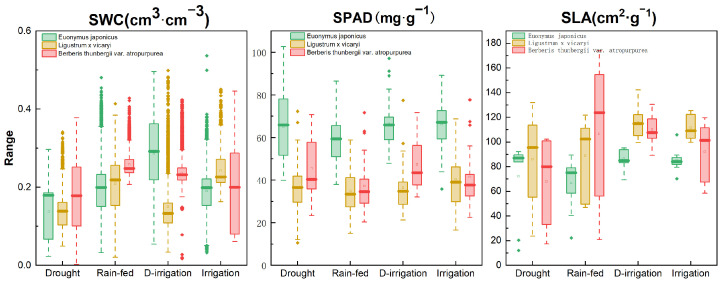
Changes in soil water content (SWC), chlorophyll content (SPAD), and specific leaf area (SLA) of three plants under different water treatment conditions.

Different moisture treatments significantly affected soil moisture content, chlorophyll content, and specific leaf area, while there were also differences in the response of different tree species to moisture (Figure 4). Soil water content increased with increasing water treatments, and the SWC was higher under the fully irrigated treatment than other treatments. The SPAD values of *E. japonicus* were higher in all treatments (38 to 102.7), whereas the SPAD values of *E. japonicus* and *B. thunbergii* var. *atropurpurea* were relatively lower (15 to 71.7; 10.6 to 77.4), especially in the drought and natural rain-fed treatments. The SPAD of the three shrubs was highest (16.6–89.2) under the fully irrigated treatment and lowest (10.6–102.7) under the drought treatment, indicating the important effect of moisture conditions on chlorophyll content. SLA values were significantly higher (*p*-value < 0.05) under the fully irrigated treatment (60.18 to 125.5) and lowest (12.13 to 17.45) under the drought treatment, indicating that the moisture conditions had significant effects on leaf structure and function.

**Figure 5:**
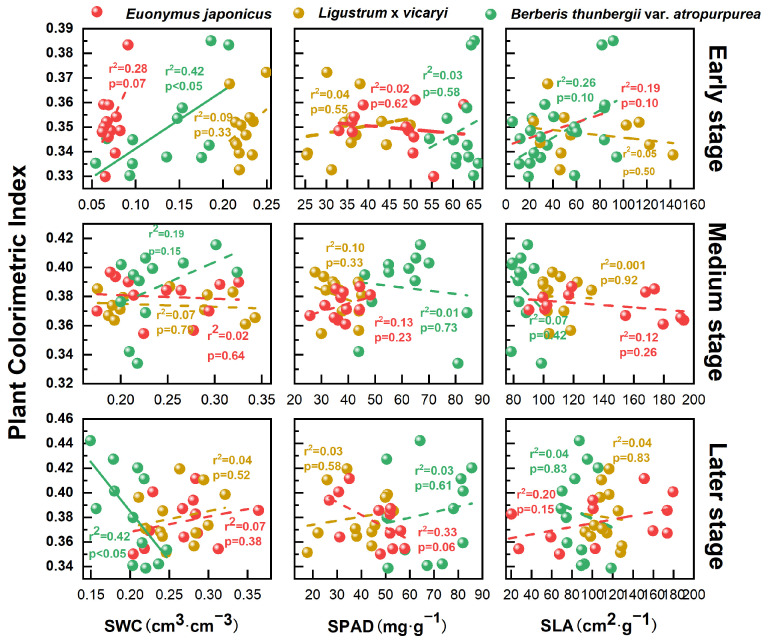
Relationship between plant colorimetric index and influence factors (SWC: soil moisture content; SPAD: chlorophyll content; SLA: specific leaf area). The solid line indicates a statistically significant (0.05) linear relationship, and the dashed line indicates a non-significant relationship.

In the early stage of the growing season, the color index of *E. japonicus* was positively correlated with the SWC (r^2^ = 0.42, *p* < 0.05), the color index of *L.* × *vicaryi* had a weaker correlation with the SWC (r^2^ = 0.07, *p* = 0.79), and *B. thunbergii* var. *atropurpurea* had a weaker correlation with the SWC (r^2^ = 0.17, *p* = 0.19); this suggests that, in the early stage, the soil moisture content and the chlorophyll content had significant effects on plant coloration indices, emphasizing the importance of moisture management in maintaining coloration in the early stages of plant growth. In the late growing season, the correlation of the color index was stronger for *E. japonicus* (r^2^ = 0.42, *p* < 0.05), whereas the correlation of the color index with the SWC was weaker for *L.* × *vicaryi* and *B. thunbergii* var. *atropurpurea* in both the mid- and late-periods. The colorfulness indices of the three plants did not show a significant relationship with either the SPAD or SLA at all three stages (Figure 5). The relationship between plant color indices and impact factors differed significantly across growth stages, suggesting that plants have different sensitivities to environmental factors at different growth stages.

The mixed-model ANOVA revealed highly significant main effects of season (*p* < 0.001, p^2^ = 0.207) and treatment (*p* < 0.001, p^2^ = 0.109) on the plant colorimetric index, whereas species had no significant effect (*p* = 0.210). Post hoc Tukey’s HSD tests demonstrated that the colorimetric index was significantly higher in the medium stage than in both the early stage (*p* < 0.001) and later stage (*p* < 0.001), with no significant difference between spring and winter (Table 1).

## 3. Discussion

### 3.1. Application of Shrub Color Index in Plant Drought-Tolerance Evaluation

In this study, we revealed the potential of color coordinates in the evaluation of plant drought tolerance by quantifying the dominant color chromaticity indices (G_CC_, Y_CC_, and R_CC_) of three greening shrubs (*E. japonicus*, *L.* × *vicaryi*, and *B. thunbergii* var. *atropurpurea*) under different moisture treatments. The results showed that drought stress significantly reduced the G_CC_, Y_CC_, and R_CC_ values of the three plants (Figure 2), with the G_CC_ of *E. japonicus* showing the largest decrease (by about 40%) under the 2023 drought conditions, and the R_CC_ of *B. thunbergii* var. *atropurpurea* showing a decrease of up to 35% in the extreme drought of 2022. This result is similar to that of Huete et al. (2002), who found that drought stress leads to changes in the spectral reflectance characteristics of plant leaves—which in turn affects color expression—using the Enhanced Vegetation Index (EVI) [24]. Using hyperspectral imaging, Dao et al. (2021) also found that drought stress leads to a shift in the position of the red edge of plant leaves and a decrease in reflectance, which in turn affects the color index [25], further explaining the physical mechanism of the changes in color indices. Based on the sensitive response characteristics of color coordinates to drought stress, this study proposes that the following technological paths can be explored in the future: ① The development of a smartphone portable analysis system (e.g., PlantSnap or a customized APP) to obtain the R_CC_ values in real time by taking photographs of leaves, and combining this with a machine learning model to establish a non-destructive prediction method for anthocyanin content [26]. ② The construction of a cross-species “color-physiology” correlation database, for example, establishing an R_CC_ threshold warning system for colorful foliage plants such as *B. thunbergii* var. *atropurpurea*. This scheme can transform the color dimensions (from G_CC_ to Y_CC_/R_CC_) expanded in the current study into a conservation decision-making tool, and suggest water replenishment or shade measures when the monitoring value is abnormal, which has significant advantages in the conservation of valuable plants—such as old and valuable trees—compared with traditional destructive sampling methods. This theoretical framework opens up new perspectives for the evaluation of drought resistance in urban greening plants, and especially provides methodological support for the precise maintenance of multicolored foliage landscape plants.

### 3.2. Adaptation Strategies of Greening Shrubs to Water Stress

This study confirmed that the shrub color index can effectively reflect its drought tolerance and that different shrubs have different adaptation strategies to water stress. Changes in water availability can affect plant color expression through the SWC. However, it is worth noting that the plant color index significantly correlated with the soil water content (SWC) only in the early part of the growing season (e.g., *E. japonicus*: r^2^ = 0.54, *p* < 0.01), suggesting that water availability is critical for the early formation of leaf color, although the role of water declines in the mid-to-late growing season. This is consistent with the conclusion of Chaves et al. (2003) on the “relationship between leaf functional traits and water use efficiency” that water conditions directly affect chlorophyll synthesis and leaf morphology [27]. This also agrees with Horike et al. (2023), that “leaf spectral characteristics are dynamically related to water status”, i.e., water conditions affect color expression by regulating chlorophyll synthesis and cell expansion pressure [28]. However, unlike previous studies, the present study found that the color index of *B. thunbergii* var. *atropurpurea* was weakly correlated with the SWC (r^2^ = 0.17, *p* = 0.19), which may be related to its higher drought tolerance. For example, *B. thunbergii* var. *atropurpurea* may reduce water loss by decreasing the specific leaf area (SLA) or increasing leaf epidermal waxes (similar to the adaptive strategy of *Rosmarinus officinalis*; Pérez-Harguindeguy et al., 2013), thus maintaining a relatively stable color performance under drought conditions [29]. In addition, the SLA is the ratio of leaf area to mass, and higher SLA values are usually associated with greater photosynthetic efficiency and resource use efficiency [30]. The increase in the SLA under the adequately irrigated condition suggests that plants are able to optimize leaf structure to adapt to the environment when well watered.

Changes in the degree of color in greening shrubs can also be reflected by the SPAD values. The G_CC_ and SPAD values of *E. japonicus* decreased significantly under drought conditions (a 40% decrease in the G_CC_ in 2023), indicating that it is sensitive to drought and its chlorophyll synthesizing ability is easily inhibited; whereas the R_CC_ and SPAD values of *B. thunbergii* var. *atropurpurea* showed smaller changes (a <10% decrease), implying that it has strong drought resistance. This difference may be related to the morphological and physiological adaptation strategies of the plant. For example, *B. thunbergii* var. *atropurpurea* may maintain its water balance by increasing leaf thickness or decreasing the transpiration rate, similar to the strategies of Lavandula dentata to enhance drought tolerance by accumulating osmoregulatory substances such as proline [31] and of *Populus euphratica* to enhance drought tolerance by accumulating betaine and proline [32]. In addition, the small fluctuation in color index (coefficient of variation < 15%) under the three moisture treatments of *L.* × *vicaryi* may be related to its leaf structure (e.g., stomatal regulation capacity) or the accumulation of osmoregulatory substances, which is similar to the mechanisms of Quercus ilex to maintain moisture under drought by adjusting stomatal conductance and of Arabidopsis thaliana to regulate stomatal closure under drought through the ABA signaling pathway. The mechanism of regulating stomatal closure is similarly analogous [33,34]. While the SPAD and SLA indices provide valuable physiological insights, our findings indicate they alone are insufficient for guiding urban shrub irrigation. Integrating color indices with SPAD/SLA provides a more robust framework for precision irrigation in urban landscapes.

### 3.3. Climatic and Phenological Drivers of Seasonal Color Variation

The interannual differences in colorimetric indices under adequate irrigation (Figure 1) likely reflect the climatic variability between 2022 and 2023. The higher peak values in 2023 (e.g., in the G_CC_ of *E. japonicus* and R_CC_ of *B. thunbergii* var. *atropurpurea*) align with the warmer spring temperatures recorded in North China during 2023 [8], which accelerated chlorophyll synthesis and anthocyanin accumulation. Notably, the synchronized color index peak across all genotypes around the day of year (DOY) 150 (late May) in 2023 corresponds to a critical phenological phase: the transition from spring leaf expansion to summer maturation. During this period, optimal temperature (15–25 °C) and photoperiod conditions (annual sunshine: 2000–2800 h) in the region (Section 4.1) converge to maximize photosynthetic pigment production, overriding species-specific traits. This synchronicity mirrors broader patterns in temperate ecosystems where photoperiod and thermal thresholds jointly regulate leaf development [22,23]. The attenuated 2022 peaks may result from episodic cold fronts or precipitation anomalies (annual rainfall: 483.9 mm ± 24% interannual variability), highlighting climate-mediated constraints on ornamental color expression.

### 3.4. Research Limitations and Outlook

Although this study provided important insights into the effects of water stress on the color index of greening shrubs, there are still some limitations and directions for future research. First, this study focused on several specific greening shrub species, and future research could be extended to a wider range of plant species to verify the generalizability of this study’s findings. For example, Carter and Knapp [35] showed that there were significant differences in the leaf optical properties of different plant species in response to water stress, suggesting that we need to cover a more diverse range of plant species in future studies to fully understand the mechanisms of the effects of water stress. Second, this study was conducted mainly under controlled conditions, and future studies could be conducted in natural environments to better understand the effects of water stress on the color index of greening shrubs. A study by Gamon and Surfus [36] pointed out that experimental results under controlled conditions may differ from the actual situation in natural environments, and so validation within natural environments is necessary. In addition, the combination of remote-sensing technology and big data analysis can be used to assess the moisture status and color changes in urban greening shrubs more comprehensively. Zhang et al. [37] successfully monitored the vegetation phenology pattern using MODIS remote-sensing data, which provided a technical reference for the dynamic monitoring of moisture status in urban greening. A similar approach can be applied to the study of water stress in urban greening shrubs to improve the temporal and spatial resolution of the data. Finally, different irrigation treatments significantly affected the soil moisture, chlorophyll content (SPAD), and specific leaf area (SLA), with distinct responses observed across shrub species. The soil water content increased proportionally with irrigation intensity, while the SPAD values peaked under full irrigation, indicating optimal chlorophyll health. The SLA also rose substantially with improved water availability, reflecting enhanced leaf structural integrity and function. These results underscore the critical need to align plant selection with site-specific water regimes in urban landscapes. The practical applications include the following: (1) Species-specific irrigation protocols: drought-sensitive shrubs (e.g., *Hibiscus syriacus*) require full irrigation to maintain chlorophyll vitality and leaf function, while drought-tolerant species (e.g., *Nerium oleander*) may thrive under reduced watering, conserving resources. (2) SPAD/SLA as diagnostic tools: monitoring the SPAD and SLA values offers a rapid, non-destructive method to assess plant water stress and inform irrigation scheduling in urban green spaces. (3) Climate-resilient landscaping: prioritizing species with a low SLA (thicker leaves) in water-scarce regions can improve urban greening sustainability as these traits correlate with reduced water loss and greater drought resilience. Future research could also explore the effects of water stress interacting with other environmental factors (e.g., light, temperature, and soil conditions) on the color index of greening shrubs. Chaves et al. [38] showed that the interaction of water stress with environmental factors such as salt stress and light significantly affects photosynthesis and the physiological status of plants. This will help reveal the adaptation mechanisms of plants under complex environmental conditions and provide a more comprehensive scientific basis for urban greening management. In recent years, the progress of research on the color variation in plants has shown that, in addition to leaves, the color variation in flowers, fruits, stems, and the other organs of plants is also affected by similar factors but with more pronounced interspecific variability [39]. For example, a study by Gould [40] pointed out that there were significant interspecific differences in the distribution of anthocyanins in different plant organs and their effects on color changes. Therefore, in future work, attention should also be paid to the study of color variations in other organs using color indices in addition to leaves for a more comprehensive understanding of plant response mechanisms to environmental stresses.

## 4. Materials and Methods

### 4.1. Study Site

The experimental study area was set up in the resource nursery of the Institute of Forestry and Pomology, Beijing Academy of Agriculture and Forestry Sciences, which is located in the vicinity of Minxi Bridge within the West Fifth Ring Road of Beijing. Geographic coordinates: 39°59′35″ N, 116°13′13″ E; altitude: about 88 m; total area: 13.33 hm^2^. The climate type for the Northern Hemisphere, a temperate continental monsoon climate, has four distinct seasons: a dry, windy spring with high evapotranspiration; a hot, rainy summer, with average annual sunshine hours of 2000~2800 h, an average daily temperature of 15~25 °C, and a relative humidity of 70~80%; an annual frost-free period of 180~200 d; an average annual precipitation of 483.9 mm; and a seasonal distribution of precipitation that is uneven—80% of the annual precipitation is concentrated in the months of June, July, and August. The soil in the experimental area was clay loam, with a bulk density of 1.46 g·cm^−3^, a maximum field water holding capacity of 30.58%, a total nitrogen content of 1.07 g·kg^−1^, a total phosphorus content of 0.96 g·kg^−1^, a total potassium content of 18.55 g·kg^−1^, and an organic matter content of 23.49 g·kg^−1^.

### 4.2. Experimental Materials

In this experiment, the main greening shrub species commonly used in green belts in North China were selected, including *Euonymus japonicus*, *Ligustrum* × *vicaryi*, and *Berberis thunbergii* var. *atropurpurea*. Plants of similar growth, 5 years old, with average plant heights of 75.4 ± 8.3 cm, 86.6 ± 6.4 cm, and 69.4 ± 7.3 cm and average crown widths of 53.7 ± 5.7 cm, 78.4 ± 7.6 cm, and 73.0 ± 12.7 cm, respectively, were selected as the experimental materials for this study, and soil conditions were kept essentially the same.

### 4.3. Experimental Design

Irrigation Experimental Design and Sample Layout: In this study, with reference to local historical meteorological data, the critical period of plant growth from May to September was divided into three phases, namely, the early (1 April to 31 May), middle (1 June to 31 July), and late (1 August to 30 September) phases. Irrigation and drought treatments with different moisture conditions were conducted during two-month periods at different times. The experiment spanned two years (2022–2023). The 2022 trial used 3 shrubs, 3 experimental periods, 3 treatments, and 3 replications for a total of 81 test plots. Based on the 2022 results, it was hypothesized that there may be a superior irrigation strategy between rain-fed and fully irrigated; therefore, a deficit irrigation treatment was added in 2023. The 2023 trial was implemented on the same three shrub species (consistent with 2022), and comprised four treatments, three experimental periods, and three replications, with a total of 108 test plots. Shrubs used in the 2023 deficit irrigation treatment were individuals planted at the same time as the 2022 trial shrubs, with the same growing conditions and specifications, and the specific modes of each treatment were implemented as follows:

Irrigated Group: Irrigation occurred in the early morning from mid-April to the end of September, combining man-made and natural precipitation. If there was no natural rainfall, then plants were fully irrigated every 10 d to ensure sufficient water supply. The duration of the drip irrigation was about 10 h, with 10 L/tree.

Deficit Irrigation Group: The irrigation time was the same as the fully irrigated group, with 5 h of drip tubing and 5 L/plant.

Rain-Fed Group: Grown under completely natural rain-fed conditions, not dependent on artificial irrigation.

Extreme Drought Group: The rain-reduction device in this study refers to the widely used rain shelter design employed in related studies [41], which is a transparent polycarbonate plastic sheet with a thickness of 2 mm and a specification of 3.0 m × 2.1 m above a galvanized stainless steel pipe trellis with a rain shelter roof (with a transmittance rate of up to 90%). The roof of the rain-reduction treatment group was fully closed, intercepting all the rainwater that was discharged to the sample outside. Among them, the roof was facing upwind at an angle of about 25°, and the heights of the roofs in the upwind and downwind directions were about 1.2 m and 1.5 m, respectively; a buffer zone of 40 cm was set in the sample square, and only the central area—with a diameter of 1.2 m—was observed and sampled to minimize the edge effect [42].

Layout of Experimental Sample Plots: The irrigation plots in this study were circular, 1.2 m in diameter, with each shrub seedling located in the center—isolated from outside water conditions by plastic water barriers 50 cm belowground and 20 cm aboveground—and watered within the plot unless under drought treatment. An 80 cm buffer zone was created between plots to minimize the edge effect. Since the area of the barrier ring was larger than the root zone of these young trees, the effect on the root system of the shrubs was less. The experiment involved three shrubs, three experimental phases, three treatments, and three replications, totaling 81 test plots [43] (Figure S1).

### 4.4. Determination and Calculation of Leaf Color Parameters

**Figure 6:**
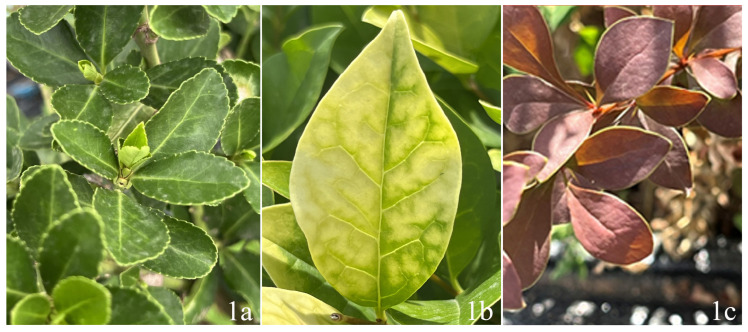
Photographs of leaves of three experimental shrub plants: (**1a**) *Euonymus japonicus*; (**1b**) *Ligustrum* × *vicaryi*; and (**1c**) *Berberis thunbergii* var. *atropurpurea*.

Plant canopy images were acquired under consistently overcast conditions (diffuse light) between 10:00 and 14:00 local time to minimize the effect of the sun’s angle (Figure 6). Each year during the growing season, photographs were acquired at ten-day intervals using a phenological camera (XST-PhotoNet-RGB, Xingshitu, Beijing, China) in a fixed position on one side of the plant, at a height of about 1 m above the ground so that the plant fills the frame of the shot. Photographs were acquired in R language software (4.3.1) (R Core Team, 2023) using the phenopix package [44] with an automatic HSV (hue–saturation–value) thresholding algorithm to separate vegetation pixels from the background (soil, equipment). The hue range was set to 50–180° (green–yellow–red spectrum), saturation > 0.1, and value < 0.95 to exclude bright non-vegetation objects; then, a binary mask generated by segmentation was applied to each image, retaining only the vegetation pixels for analysis; finally, digitally numbered (DN) values were extracted for each vegetation pixel in the red (R), green (G), and blue (B) channels to reflect the plant seasonal dynamics of leaf colorimetry.

The main colors of the leaves of the three shrubs in this study were green (*E. japonicus*), red (*B. thunbergii* var. *atropurpurea*), and yellow (*L.* × *vicaryi*); therefore, the color coordinates of green (G_CC_), red (R_CC_), and yellow (Y_CC_) were extracted for each of the three shrubs in the following way:

Vegetation Color Index Extraction: From the digital color values of each image, the green color coordinate (G_CC_) was calculated. The G_CC_ is a vegetation index derived from photographic images that quantifies the degree of greenness relative to the total luminance. The G_CC_ was calculated using the following formula: G_CC_ = G_DN_/(R_DN_ + G_DN_ + B_DN_).

The G_DN_, R_DN_, and B_DN_ are green, red, and blue numbers, respectively [45]. Similarly, red and yellow color coordinates (R_CC_ and Y_CC_) were calculated. In this case, the Y_CC_ was calculated using one-half of the sum of the G_CC_ and R_CC_.

### 4.5. Determination of Soil Water Content

A soil moisture sensor (EC-5) of the ZL6 soil moisture monitoring system (Pullman, WA, USA) was installed in one of the three replicate plots of each shrub species for each irrigation treatment to continuously and automatically determine the dynamic changes in soil moisture at a 10 cm depth at 30 min intervals. For all other replicate plots, soil moisture was manually measured discontinuously using a handheld Delta-T Devices WET-2 sensor (Delta-T Devices Ltd., Cambridge, UK) at 10-day intervals during the experimental periods.

### 4.6. Response Ratio

In plant stress research, the response ratio (RR) is a commonly used metric for comparing measurements under two conditions and can serve as an indicator of differences in plant performance under the fully irrigated condition versus drought, rain-fed, and deficit irrigation conditions. Typically, a response ratio greater than 0 indicates an increase under the comparison conditions, and less than 0 indicates a decrease. It is calculated as:

RR = ln(B_s_/B_ns_) (1)

where B_s_ is the color index of the plants under drought, rain-fed, and deficit irrigation conditions; and B_ns_ is the color index of plants under the fully irrigated condition.

### 4.7. Statistical Analysis

Differences between coloration indices of different shrub species under different degrees of drought stress were analyzed using repeated-measures analysis of variance (ANOVA), and correlations between physiological indices of shrubs were analyzed using Pearson’s correlation analysis. Statistical analyses were performed in SPSS 27.0 (IBM, Inc., Armonk, NY, USA) and Microsoft Excel (Redmond, WA, USA, 2003), and the graphs were generated in Origin 2021 (OriginLab, Northampton, MA, USA).

## 5. Conclusions

In this study, we systematically revealed the dynamic effects of water stress on plant ornamental properties and interspecific differences by quantifying the main color chromaticity indices (G_CC_, Y_CC_, and R_CC_) of greening shrubs. The results showed that under extreme drought conditions, the G_CC_ of *E. japonicus* decreased by 40% in the late growing season of 2023, and the R_CC_ of *B. thunbergii* var. *atropurpurea* decreased by 35% during the same period of 2022, whereas the color index of *L.* × *vicaryi* fluctuated the least (coefficient of variation < 15%) and its yellow leaf color was bluntly susceptible to moisture changes; the early part of the growing season was the critical period of moisture sensitivity, and the soil water content (SWC) of *E. japonicus* significantly and positively correlated (r^2^ = 0.42, *p* < 0.05) but the correlation of *B. thunbergii* var. *atropurpurea*—due to drought-resistant mechanisms (e.g., a reduction in the specific leaf area, an increase in epidermal wax) was weak (r^2^ = 0.17). Under the deficit irrigation condition (SWC ≈ 40%), *B. thunbergii* var. *atropurpurea* was still able to maintain an R_CC_ > 0.8, whereas *Euonymus japonicus* required an SWC > 60% to maintain a high G_CC_, highlighting the former’s advantage in water-saving greening. The drought-resistant mechanisms of the tree species differed significantly: the chlorophyll synthesis of *E. japonicus* was easily inhibited by drought, *L.* × *vicaryi* stabilized the anthocyanin content through morphological and physiological regulation, and *B. thunbergii* var. *atropurpurea* relied on stomatal regulation or osmotic-substance accumulation to buffer the water fluctuation. The color indices (G_CC_, R_CC_, and Y_CC_) not only have high sensitivity (e.g., the G_CC_ response ratio of *E. japonicus* reaches −0.20) but also can assess the drought resistance of colorful foliage plants in multiple dimensions, and their application thresholds (e.g., the R_CC_ of *B. thunbergii* var. *atropurpurea* is >0.8, corresponding to a SWC ≈ 40%) can provide quantitative bases for the screening of plants in arid zones (prioritizing the *B. thunbergii* var. *atropurpurea*) and precise irrigation (*E. japonicus* needs high-frequency water replenishment); this can be further optimized for the sustainable management of urban ecological landscapes through integration with remote-sensing technology.

## Figures and Tables

**Table 1:** Analysis of variance (ANOVA) results for the effects of species, season, and treatment (fixed factors) on plant colorimetric index.

Source	df	MS	F-Value	*p*-Value	Partial η^2^
**Species**	2	0.001	1.566	0.210	0.008
**Season**	2	0.045	53.859	**<0.001**	**0.207**
**Treatment**	3	0.014	16.890	**<0.001**	**0.109**

Notes: replicate was included as a random factor. MS: mean squares; Partial η^2^: partial eta-squared.

## Data Availability

The data that support the findings of this study are available on request from the corresponding author.

## Supplementary Materials

The following supporting information can be downloaded at https://www.mdpi.com/article/10.3390/plants14152253/s1, Figure S1: Experimental diagram of the shrub planting. The size of each plot was a circle with a radius of 60 cm, with 2 m between each shrub.
