# Supplementary figures and images for: RGB Imaging and Irrigation Management Reveal Water Stress Thresholds in Three Urban Shrubs in Northern China
Source: Plants (Basel). 2025 Jul 22;14(15):2253. doi: 10.3390/plants14152253 (PMC12348786; doi:10.3390/plants14152253)

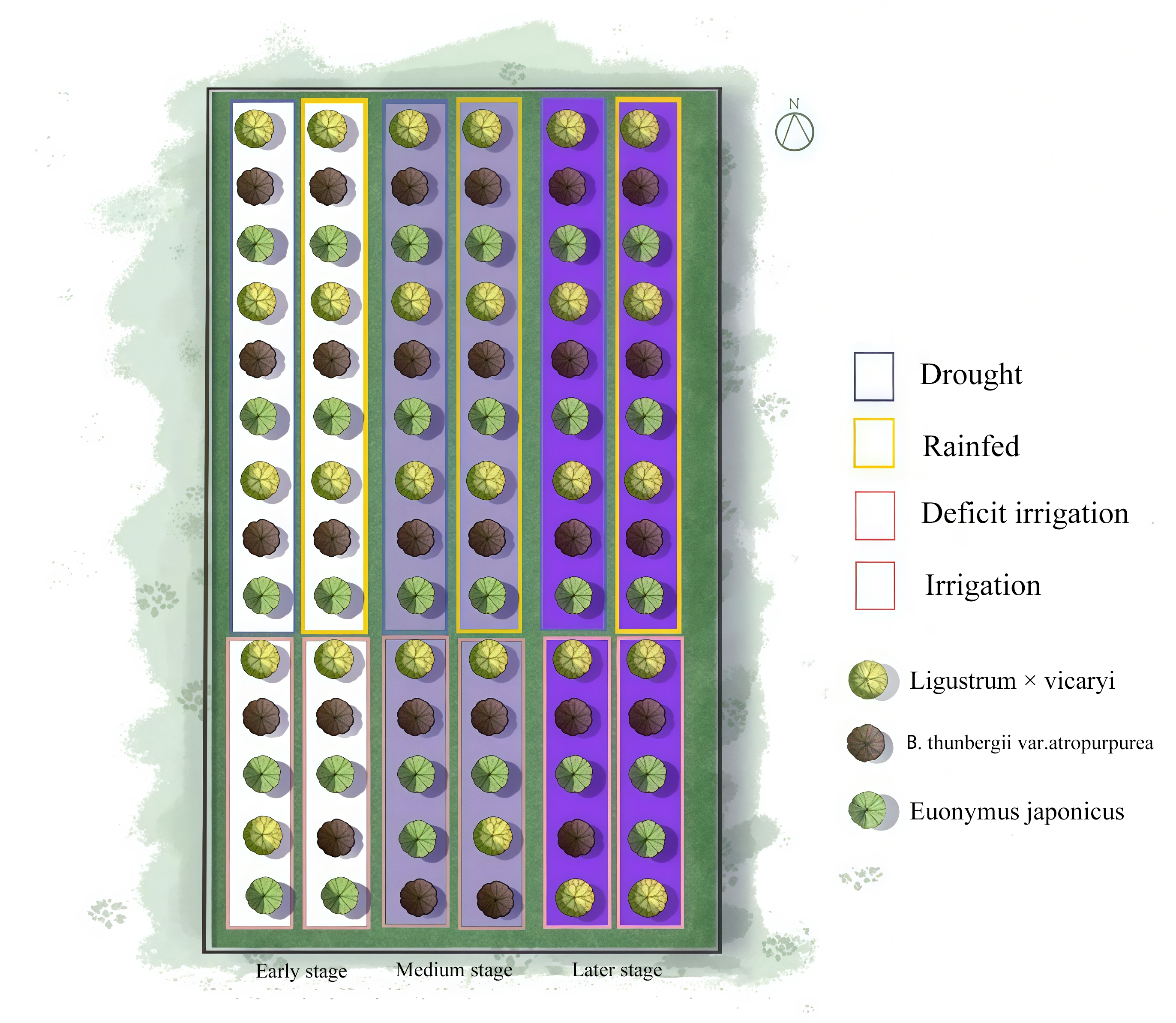

Supplement: Supplementary file 1 [file plants-14-02253-s001.zip › plants-3681477-supplementary.jpg]
